# *Salmonella* enhances osteogenic differentiation in adipose-derived mesenchymal stem cells

**DOI:** 10.3389/fcell.2023.1077350

**Published:** 2023-03-15

**Authors:** Nuradilla Mohamad-Fauzi, Claire Shaw, Soraya H. Foutouhi, Matthias Hess, Nguyet Kong, Amir Kol, Dylan Bobby Storey, Prerak T. Desai, Jigna Shah, Dori Borjesson, James D. Murray, Bart C. Weimer

**Affiliations:** ^1^ Department of Animal Science, College of Agricultural and Environmental Sciences, University of California, Davis, Davis, CA, United States; ^2^ Department of Population Health and Reproduction, 100K Pathogen Genome Project, Davis, CA, United States; ^3^ Department of Pathology, Microbiology and Immunology, University of California, Davis, Davis, CA, United States

**Keywords:** pathogenic and infectious disease, apoptosis, cell death, host/bacteria interactions, MSC

## Abstract

The potential of mesenchymal stem cells (MSCs) for tissue repair and regeneration has garnered great attention. While MSCs are likely to interact with microbes at sites of tissue damage and inflammation, like in the gastrointestinal system, the consequences of pathogenic association on MSC activities have yet to be elucidated. This study investigated the effects of pathogenic interaction on MSC trilineage differentiation paths and mechanisms using model intracellular pathogen *Salmonella enterica* ssp *enterica* serotype Typhimurium. The examination of key markers of differentiation, apoptosis, and immunomodulation demonstrated that *Salmonella* altered osteogenic and chondrogenic differentiation pathways in human and goat adipose-derived MSCs. Anti-apoptotic and pro-proliferative responses were also significantly upregulated (*p* < 0.05) in MSCs during *Salmonella* challenge. These results together indicate that *Salmonella*, and potentially other pathogenic bacteria, can induce pathways that influence both apoptotic response and functional differentiation trajectories in MSCs, highlighting that microbes have a potentially significant role as influencers of MSC physiology and immune activity.

## Introduction

Mesenchymal stem cells (MSCs) have a known capacity for self-renewal and differentiation into cartilage, bone, and adipose tissue (Dominici et al., 2006), making these cells of great interest in regenerative medicine research (Fossett and Khan, 2012; Marra and Rubin, 2012; Merimi et al., 2021). In addition to their renewal abilities, MSCs are also recruited *via* the secretion of paracrine factors to areas of inflammation where they exhibit immunomodulatory functions (Ortiz et al., 2003; Balaji et al., 2012). In cooperation with recruited immune cells, MSCs moderate inflammation *via* expression of anti-inflammatory cytokines (Romieu-Mourez et al., 2009; Galindo et al., 2011; Roddy et al., 2011), inhibit T-lymphocyte activation, and alter macrophages to express a regulatory anti-inflammatory phenotype toward increased phagocytic activity (Balaji et al., 2012; Eggenhofer and Hoogduijn, 2012).

MSCs are recruited to and subsequently secrete anti-microbial peptides at sites of bacterial infection due to localized inflammation (Krasnodembskaya et al., 2010). Pathogenic and non-pathogenic microbes interact with MSCs at mucus membranes, where tissue turnover is high and immune-responsive cells infiltrate to control pathogens (Xu et al., 2014). The outer mucosal layer matrix of the gut lumen is one such interface that is rich with both microbes and host cells and an area where MSCs come into frequent contact with microbial inhabitants and invaders (Nagashima et al., 2017; Schroeder, 2019). Gut inflammation and the subsequent destruction of intestinal epithelial cells induces MSC recruitment to facilitate tissue recovery in the intestinal tract (Semont et al., 2006). While this apoptotic epithelial cell response to infection is well characterized (Kim et al., 1998; Wemyss and Pearson, 2019), little is known about the consequences of bacterial association on the behavior of mesenchymal stem cells.

The long-term effects of MSCs and pathogen interactions remain understudied, but there are aspects of this host cell-pathogen relationship that have been previously explored. Treatment of MSCs with the immunostimulant membrane component lipopolysaccharide (LPS) and with Gram-negative *Escherichia coli* increases osteogenesis and decreases adipogenesis, while stimulation with Gram-positive *Staphylococcus aureus* decreases osteogenesis and adipogenesis (Fiedler et al., 2013). Immune functionality of MSCs can also be altered by bacterial association, as evidenced by previous work done using invasive pathogen *Salmonella* Typhimurium (Kol et al., 2014). Intracellular association of *Salmonella* in MSCs increased transcription and secretion of immunomodulatory products *IL6* and *IL8*, but also reduced MSCs’ ability to inhibit T-cell proliferation (Kol et al., 2014). These changes in viability, immunomodulatory functions, and differentiation paths of MSCs due to bacterial association confirms that MSC-bacterial interactions not only follow a different course than epithelial cells, but also illustrates that MSCs maintain such altered functions beyond acute infection and association.

Microbial invaders, like *Salmonella,* are in part recognized by host cells *via* Toll-like receptors (TLRs). TLRs are present on many host cells and recognize surface-expressed bacterial components like LPS, alerting the triggered cell to the presence of a pathogen (Kawasaki and Kawai, 2014). MSCs, along with gut epithelial cells, express TLRs but it is unclear how pathogens regulate and interact with these receptors on MSCs as compared to epithelial cells (Hwa Cho et al., 2006; Raicevic et al., 2010). Tomchuck et al. (2008) reported the promotion of MSC migratory abilities as a result of TLR activation, whereas a study by Pevsner-Fischer et al. (2007) found TLR activation shifts lineage commitment to proliferation. TLRs play a central role in the detection of and defense against pathogens, and though important, there are also other pathogen-specific activating signals, like the type three secretion system (T3SS) in *Salmonella.*

*Salmonella* pathogenesis in epithelial cells is primarily mediated *via* the T3SS, which injects effector proteins that ultimately lead to apoptosis (Pilar et al., 2013; Rivera-Chavez et al., 2013). T3SS proteins target a variety of host cell regulators, including the NFκB pathway, creating an inflammatory environment which increases microbial internalization (Thiennimitr et al., 2012; Pilar et al., 2013). MSCs have early transcription factors, such as peroxisome proliferator-activated receptor gamma (PPARG) (Pascual et al., 2005) and secreted phosphoprotein 1 (SPP1) (Sabo-Attwood et al., 2011), that are involved in inflammatory responses but which also have a role in MSC differentiation. These transcription factors may be a bridge between microbe-induced inflammation and the cellular response of MSCs. The different responses of epithelial cells and stem cells to invasion by *Salmonella* suggest stem cells undergo distinct conditioning that results in lasting immunomodulatory changes. The microbial modulation of the immune system is an area of much interest, but the unique abilities of MSCs coupled to their physical positioning at pathogenic interfaces makes these stem cells of interest given the potential widespread impact cellular reprogramming of such a fundamental cell type could have. It is possible that microbial modulation of the immune system in MSCs provides pathogens with an uncharted method of tissue infiltration, resulting in yet undescribed biological impact.

Testing the impact of microbial association on the proliferation and differentiation of stem cells *in vitro* is resource intensive, as human sampling can be both costly and difficult to arrange. Livestock models present an invaluable option to the problem of acquiring human-derived stem cells, as livestock MSCs are typically more readily accessible than human subjects, provide larger sample quantities than small animal models, and livestock stem cells have been shown to accurately reflect the behavior of their human counterparts in many instances (Harness et al., 2022). One source of livestock-derived stem cells is goats, who share many physiological similarities with humans and whose adipose-derived MSCs have been previously confirmed as appropriate for modeling human adipose-derived MSCs (Harness et al., 2022). The goal of understanding if goat cells can be used as a translational model human studies is a goal of this strudy. In this work, both human and goat MSCs were utilized to explicate the effect of pathogen association on trilineage behavior, to assess similarities and differences in their general response to bacterial invasion/association between the species, and to further highlight the usefulness of livestock stem cells for human research with translational perspectives prior to clinical trials.

This study evaluated the effect of *Salmonella* association on the immunomodulatory behavior of human and goat MSCs. It was hypothesized that MSCs would internalize *Salmonella*, resulting in altered MSC trajectories toward pro-osteogenic commitment in conjunction with the induction of an anti-inflammatory, and altered immunological phenotypes. Mechanisms indicative of MSC survival, proliferation, and immune regulation in response to *Salmonella* interaction were evaluated to address this hypothesis. The data from this study indicates stem cells altered their therapeutic phenotypes and behavior in response to association with an intracellular pathogen, suggesting that microbial-specific alterations in MSC differentiation and inflammatory status can influence broader stem cell fate and ultimately stem cell functionality.

## Materials and methods

### Cell culture

Human adipose-derived mesenchymal stem cells (hASCs) were isolated in the laboratory of Dr. Dori Borjesson (University of California, Davis) and cultured in Minimum Essential Medium Alpha Modification (MEM-α, HyClone Laboratories, Logan, UT) with 20% fetal bovine serum (FBS, HyClone) and 1% penicillin-streptomycin (P/S, Gibco Life Technologies). Goat adipose-derived mesenchymal stem cells (gASCs) were isolated in the laboratory of Dr. Matthew Wheeler (University of Illinois, Urbana-Champaign), as described by Monaco et al. (2009), and expanded on as described by Mohamad-Fauzi et al. (2015). ASCs were cultured in 5% CO_2_/37°C, and used at passage six. Colonic epithelial cells (Caco-2; ATCC HTB-37) were obtained from American Type Culture Collection (Manassas, VA) and grown according the method defined by Shah et al. (2014).

### Bacteria culture

*Salmonella enterica* ssp *enterica* serotype Typhimurium LT2 (ST), 14028S, serotype Enteritidis (BCW_4673), serotype Saint Paul (BCW_88) and serotype Newport (BCW_1378) were grown in Luria-Bertani (LB) broth (Teknova, Holister, CA) and incubated with shaking (200 rpm) at 37°C. Bacterial cultures were grown according to the method in Kol et al. (2014) for this study. Multiple serotypes were used to account for sero-diversity and assess if these organisms induced different responses as previously observed (Kol et al., 2014).

### Quantification of microbe association

Association was determined using the gentamicin protection assay (Elsinghorst, 1994) and modified from Kol et al. (2014) as follows: ASCs were plated (4 × 10^4^) in a 96-well plate and incubated overnight; bacteria were suspended in serum-free medium (10^8^ CFU/mL) and added to the ASCs with a multiplicity of infection (MOI) 1:100 (MSC:bacteria).

### Transmission electron microscopy

hASCs were plated on glass slides (Nalge Nuc International, Naperville, IL) and incubated for 2 h with ST (Kol et al., 2014). Preparation and completion of transmission electron microscopy (TEM) was conducted as outlined in Kol et al. (2014).

## Differentiation

Adipogenic and osteogenic differentiation was done using ASCs in 6-well plates at 2.5 × 10^5^ ASCs/well and incubated with ST for 1 h as described above. Chondrogenic differentiation was done in T-25 flasks at 3 × 10^5^ ASCs/flask and incubated with ST for 1 h as described above. Following treatment with gentamicin, ASCs were washed with PBS to remove bacteria from the suspension and subsequently cultured for 48 h in expansion medium to 70%–80% confluence, after which differentiation medium was added.

### Osteogenic differentiation assay

ASCs were cultured in osteogenic medium, fixed, rinsed and visualized under light microscopy as described in Mohamad-Fauzi et al. (2015). hASCs were cultured for 14 days, whereas gASCs were cultured for 21 days. Control non-induced cells were cultured in expansion medium.

### Chondrogenic differentiation assay

Chondrogenic differentiation was carried out as described by Zuk et al. (2001). Following ST incubation, 70%–80% confluent cells were trypsinized and suspended in expansion medium for 14 days, then processed and visualized as described by Mohamad-Fauzi et al. (2015).

### Adipogenic differentiation assay

Cells were cultured for 21 days in adipogenic induction medium, fixed, stained, and visualized according to the methods described in Mohamad-Fauzi et al. (2015).

### RNA extraction and cDNA synthesis

ASCs were flash frozen prior to RNA extraction. For analysis of immunomodulatory factors, ASCs were plated in 6-well plates (3 × 10^5^ cells/well) and incubated with ST as described above. LPS (Sigma) was added at 10 ng/mL. MSCs were washed with PBS, and immediately lysed with TRIzol Reagent (Life Technologies) as described previously (Chen et al., 2017) Total RNA was extracted as described by Mohamad-Fauzi et al. (2015). Total RNA (1 µg) was used for first-strand cDNA synthesis using SuperScript II Reverse Transcriptase (Life Technologies) and oligo-dT primers according to the manufacturer.

### Quantitative RT-PCR

Primers (Supplementary Tables S1, S2) were designed using Primer3 when not directly obtained from references. All primers spanned exon junctions or included introns. mRNA expression was quantified using Fast SYBR Green reagent (Life Technologies) on the Bio-Rad CFX96 platform (95°C for 20 s, 40 cycles of 95°C for 3 s, and 60°C for 30 s), followed by melt curve analysis. Gene expression was normalized to GAPDH using 2^−ΔΔCT^ (Livak and Schmittgen, 2001; Schmittgen and Livak, 2008). Differences in differentiation gene expression were calculated as fold-changes relative to cells cultured in expansion medium (non-induced) and not treated with bacteria (non-treated). Inflammatory gene expression was calculated as fold-changes relative to non-treated control cells. Treatments were analyzed in pairwise comparisons using the Student’s *t*-test on JMP software (SAS Institute) (*p ≤* 0.05). Data are presented as mean ± SEM with three biological and technical replicates.

### GeneChip expression analyses

Caco-2 infection samples with *Salmonella* LT2 were conducted using Affymetrix HGU133Plus2 GeneChip. Custom arrays containing all annotated coding and intergenic sequences of *S. enterica* spp. *enterica* sv Typhimurium LT2 (Marcobal et al., 2011; Ng et al., 2013; Shah et al., 2013). Data were normalized using MS-RMA (Stevens, 2008) and analyzed using Significance Analysis of Microarrays (SAM) (Champine et al., 2007; Ng et al., 2013).

### hASC RNA sequencing

Total RNA (1 µg) from hASCs was used to construct sequencing libraries with the Truseq Stranded Total RNA LT Kit (Illumina) as described previously (Chen et al., 2017; Haiminen et al., 2019; Beck et al., 2021). Quality of RNA and constructed libraries was determined *via* 2100 Bioanalyzer. Libraries sequenced using an Illumina HiSeq 2000 (BGI@UC Davis, Sacramento, CA) with single-end 50 bp. Reads were aligned using the UCSC hg19 human reference genome (ftp://igenome:G3nom3s4u@ussd-ftp.illumina.com/Homo_sapiens/UCSC/hg19/Homo_sapiens_UCSC_hg19.tar.gz) and annotated using “-a 10 –b2-very-sensitive -G”. Read count and normalization was done using Cufflinks package (version 2.2.0) with flags “-u -G”. Tables from cuffnorm and cuffdiff imported into Ingenuity Pathway Analysis (IPA; Ingenuity Systems, version spring 2014). Sequence quality was examined using Phred.

### Ingenuity Pathway Analysis

IPA (version 01-20-04) was used to determine biological pathways associated with gene expression profiles. Networks represent molecular interaction based on the IPA knowledge database. Estimation of probable pathway association was determined Fisher’s exact test, and predicted direction change was decided by the IPA regulation z-score algorithm (z-score ≥ 2 and ≤ 2 means a function is significantly increased or decreased, respectively) (St-Pierre et al., 2013).

## Results

### Microbial association with adipose-derived mesenchymal stem cells

**FIGURE 1:**
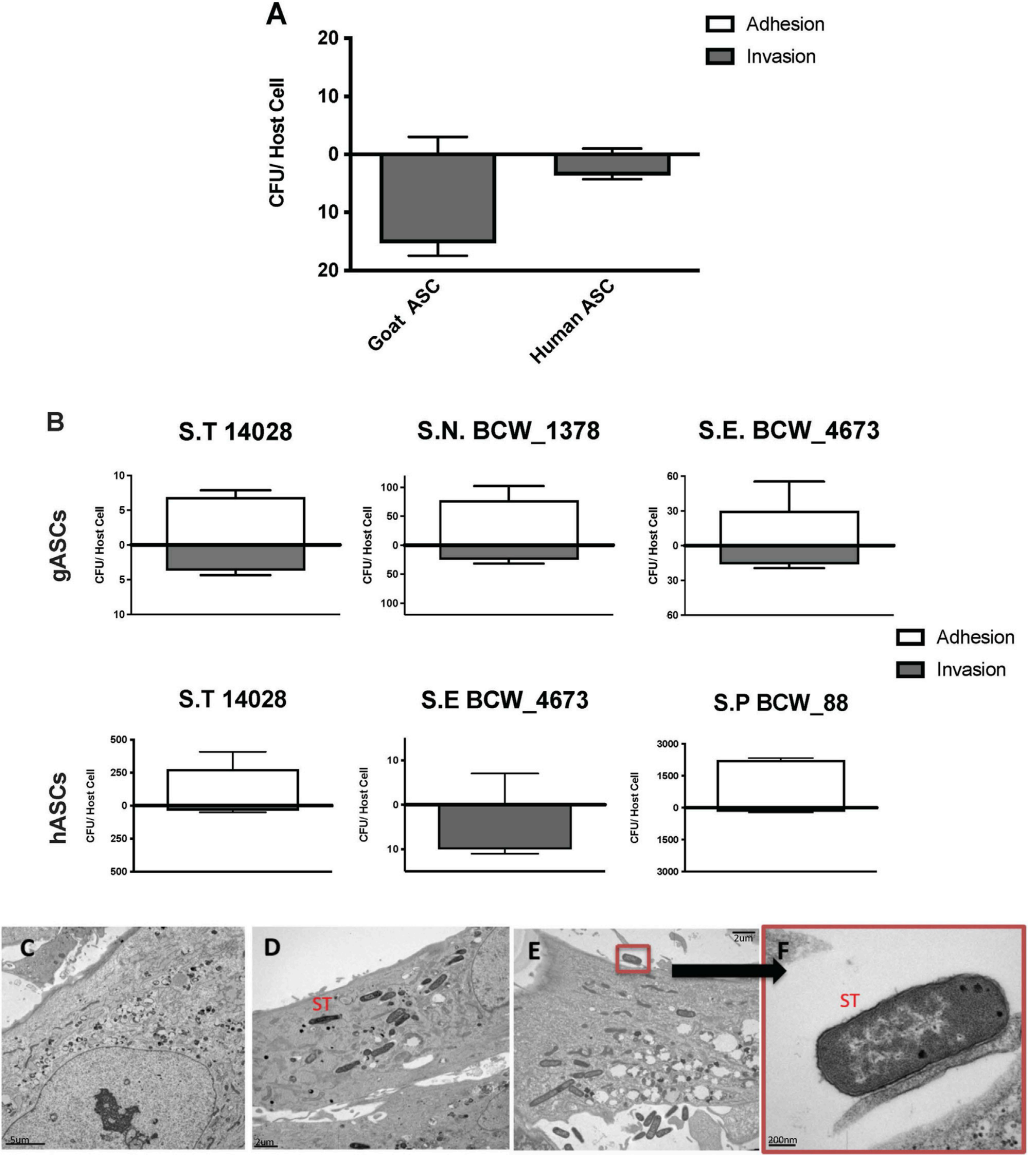
Microbial Association with human and goat ASCs **(A–F)**. ASCs presented a uniform pattern of *Salmonella enterica* ssp *enterica* serotype Typhimurium LT2 (ST) infection, the total associated bacteria were invaded, gASC show significantly higher invasion compared to human cells **(A)**. ASCs susceptibility to invasion was not exclusive to ST, association patterns were microbe specific; 35%, 12% *Salmonella enterica* ssp *enterica* serotype Typhimurium 14,028, and 25%, 100% *Salmonella enterica* ssp *enterica* serotype Enteritidis (BCW_4673) were invaded in goat and human ASCs respectively **(B)**. In gASCs, 35% *Salmonella enterica* ssp *enterica* serotype Newport (BCW_1378) and in hASCs, 7% of *Salmonella enterica* ssp *enterica* serotype Saint Paul (BCW_88) were invaded **(B)**. Intracellular ST was observed by TEM 2 h post MSC co-incubation **(D–F)**, consistent with control non-treated hASCs **(C)**, ST infected cells showed no signs of cellular toxicity **(D–F)**. ST adherence to hASC was observed at various sites **(E–F)**.

Human and goat adipose-derived mesenchymal stem cells (ASCs) were susceptible to *in vitro* infection with *S. enterica* ssp *enterica* Typhimurium LT2 (ST) (Figure 1), corroborating previous observations regarding canine ASC susceptibility (Kol et al., 2014). Though both human ASCs (hASC) and goat ASCs (gASC) were vulnerable to and displayed intracellular invasion by ST, gASCs showed significantly increased ST invasion compared to human cells (*p* = 0.006) (Figure 1A). In addition to ST, the other *Salmonella* serotypes were also able to invade ASCs, though to a lesser degree as compared to ST (Figure 1B). The ability of multiple *Salmonella* serotypes to exist intracellularly in ASCs further supports previous work by our group demonstrating this ability in multiple serotypes (Kol et al., 2014), suggesting a consistent trend of ASC vulnerability to common pathogens (Figures 1A, B).

Invasion and adhesion of hASCs by ST was further evaluated utilizing TEM 2 h post hASC-microbe co-incubation (Figures 1C, F). Intracellularly infected hASCs did not display visual markers of morphological distress or signs of apoptosis. In addition to invasion, adherence of ST to hASC cell surface was also observed; an intimate host-microbe association which is consistent with what has been seen with other non-pathogenic bacteria (Kol et al., 2014).

### ASC immunomodulation activity is altered by infection

**FIGURE 2:**
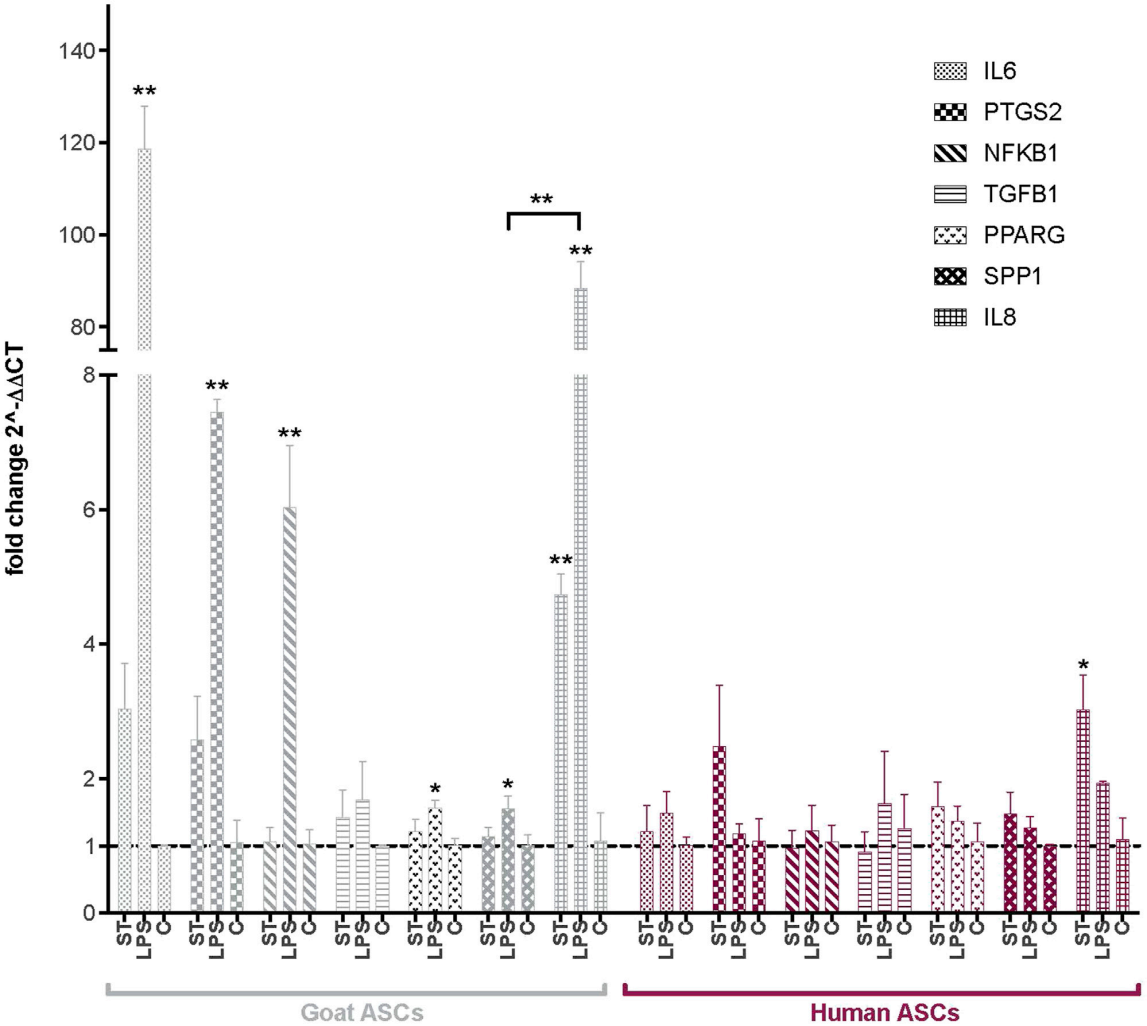
Expression of immunomodulatory factors in ASCs post-microbial association. Quantitative PCR analysis of IL6, PTGS2, NFKB1, TGFB1, PPARG, SPP1, and IL8 expression in goat and human ASCs treated with ST or LPS. Data is presented as fold change (± SEM) in relative to expression levels in non-treated cells (“C”) (fold change ∼1, indicated by the dotted line). Statistical significance of *p <* 0.05 is denoted by an asterisk (*), and *p* < 0.01 denoted by two asterisks (**). Goat cell data is presented in gray and human cell data is presented in maroon.

Following co-incubation with ST, the expression of several key immunomodulatory genes in ASCs was evaluated *via* qPCR (Figure 2). Interleukin 8 and 6 (*IL8*, *IL6*), prostaglandin-endoperoxide synthase 2 (*PTGS2*), nuclear factor of kappa light polypeptide gene enhancer in B-cells 1 (*NFΚB1*), transforming growth factor beta 1 (*TGFB1*), *PPARG* and *SPP1* were selected as markers of immunomodulatory activity in human and goat ASCs. As compared to non-infected cells, ST treated gASCs (Figure 2) and hASCs (Figure 2) both significantly increased *IL8* expression (*p ≤* 0.033, *p ≤* 0.005, respectively). No other surveyed ASC immunomodulatory markers displayed significant differences in expression as compared to the controls.

The effect of LPS treatment on these immunomodulatory genes was also evaluated as a positive control for MSC response to inflammatory markers. *IL8* expression was increased in both hASCs and gASCs in response to LPS treatment, but this increase was only significant in gASCs (*p ≤* 0.0001). LPS treatment of gASCs also induced a significant increase in the expression of *IL6* (*p =* 0.0001), *PTGS2* (*p =* 0.0009), *NFKB1* (*p =* 0.0002), *PPARG* (*p* = 0.0204), *SPP1* (*p =* 0.037), as well as *IL8* (*p* ≤ 0.0001) (Figure 2). LPS treatment of hASCs also resulted in increased expression of the evaluated immunomodulatory genes, but none were significant compared to the control hASCs (Figure 2).

### Effect of ST association on the genetic activity of ASCs

**FIGURE 3:**
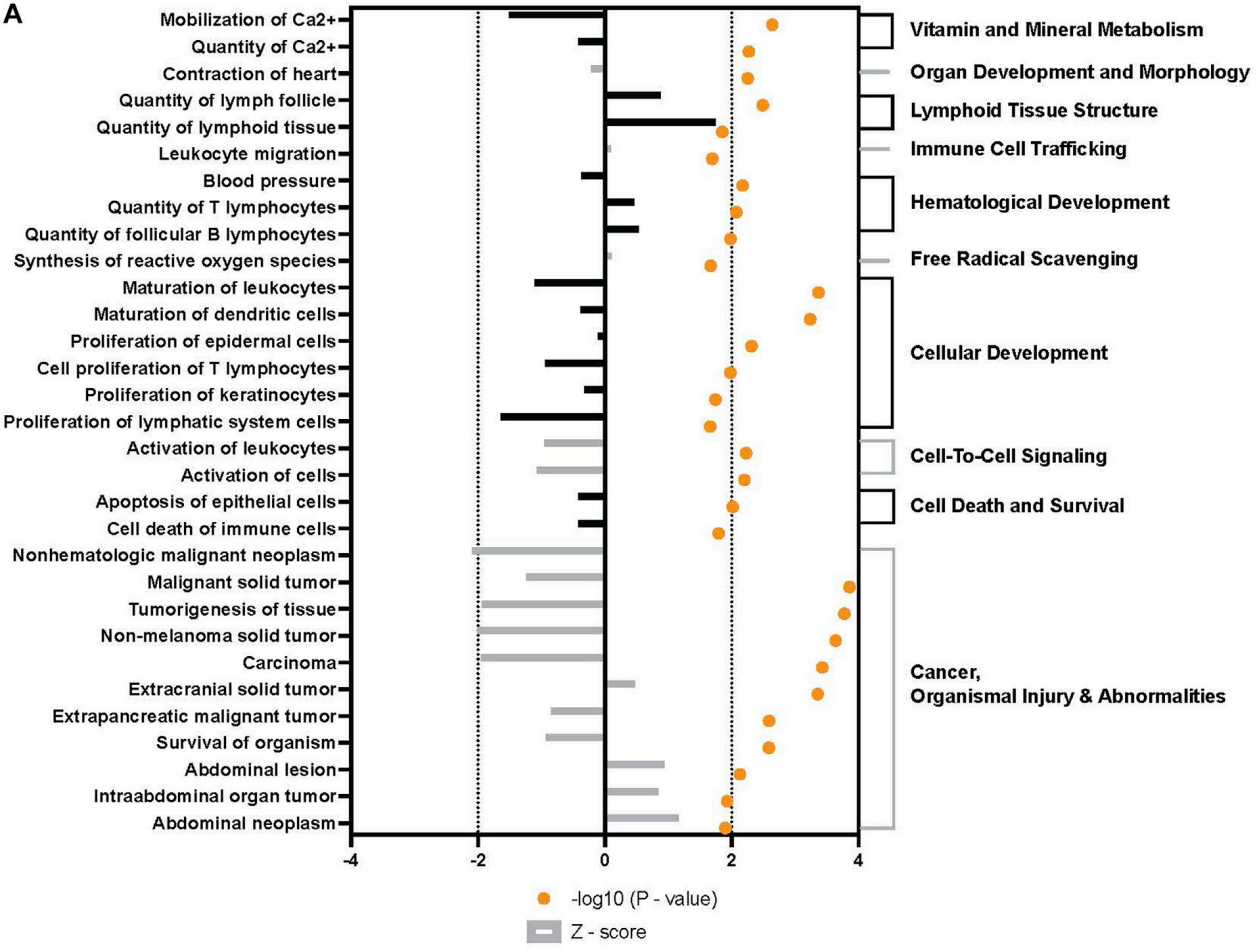
Downstream trends analysis of differentially expressed genes in hASCs post microbial challenge. The IPA regulation z-score algorithm was used to identify biological functions expected to increase or decrease based on the gene expression changes described in our dataset. Predictions base on *p*-value and z-score; positive z-score implies an increase in the predicted function, a negative z-score a decrease (z-score ≥ 2 or ≤ −2 represented by black dotted lines). *p*-values ≤0.05 (orange dots determined by Fischer’s exact test), illustrate a significant association between a given biological function and genes differentially expressed in our dataset (*p*-value ≤0.05). *p*-values are presented as log-transformed. Shapes associated with each gene name and broad category indicate the general classification of the gene product, enzyme, growth factor, etc.

Broad analysis of gene expression in hASCs co-incubated with ST found 118 significantly differentially expressed genes (*p* ≤ 0.05, FDR = 0.1). Further canonical pathway analysis of this expression data revealed infected hASCs repressed gene pathways associated with cell-to-cell signaling, cell death, and apoptosis (Figure 3).

**FIGURE 4:**
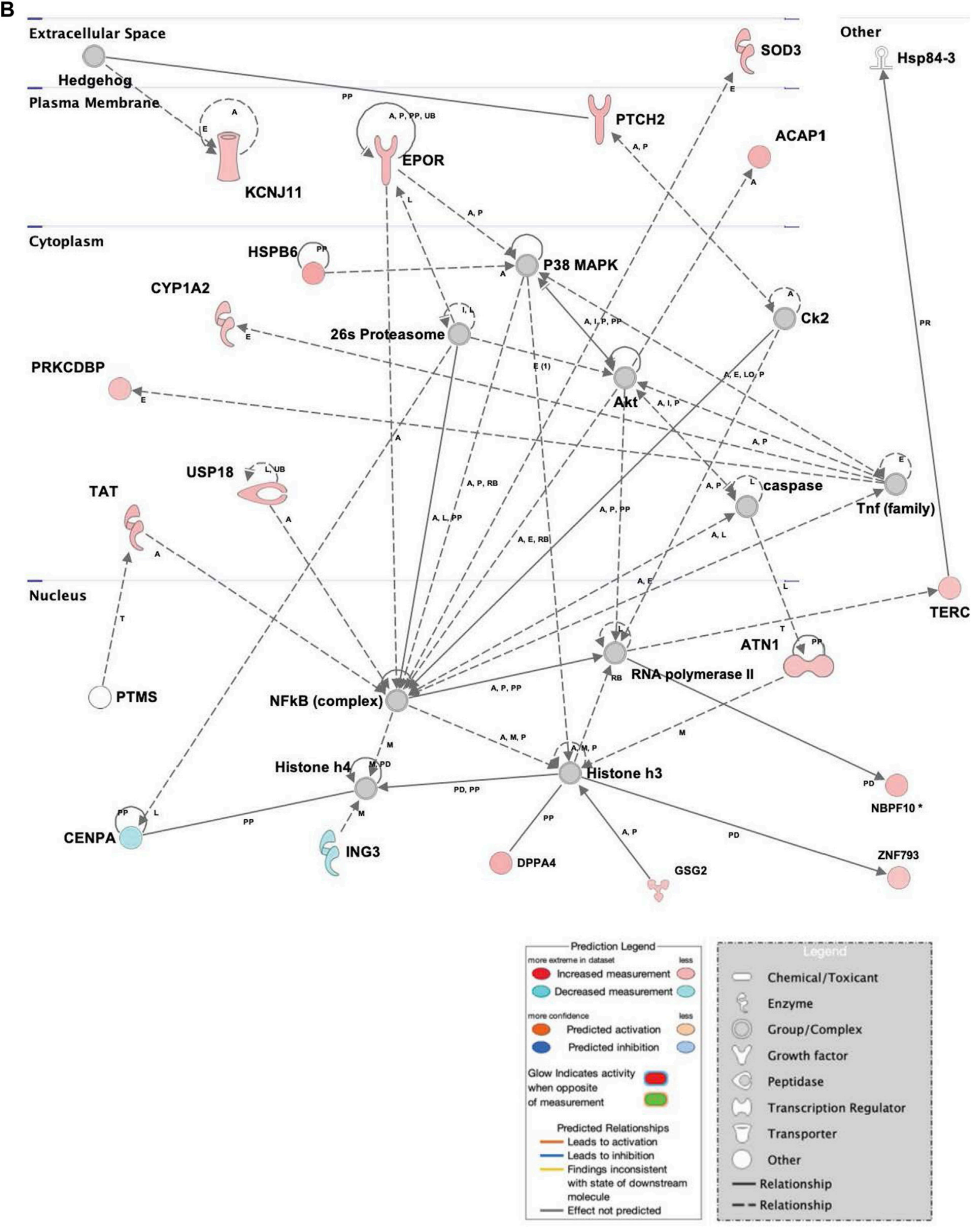
Network displays interactions between genes regulating cell signaling, cellular function and maintenance, and vitamin and mineral metabolism that were differentially expressed in hASCs treated for 60 min with S.T compared with untreated control. Upregulated genes are colored in shades of red, downregulated in shades of green (*p*-value ≤ 0.05). IPA inserted Genes in white because they are connected to this network; dashed and solid lines denote indirect and direct relationships between molecules. The IPA molecule activity predictor assessed the activity of molecules strongly connected to this network; blue and orange colored molecules are predicted to have decreased and increased activity, respectively. Shapes associated with each gene name and broad category indicate the general classification of the gene product, enzyme, growth factor, etc.

The repression of genes related to cell survival and death, as well as promotion of proliferation and multipotency in infected hASCs (Figure 4) is consistent with the observation of continued hASC viability post ST association. Expression of single genes related to cell death and viability confirms the importance of these pathways in cell persistence post-ST association. The gene for survival-related heat shock protein B6 (*HSPB6*) was upregulated and there was MAP-predicted association of *v-akt* murine thymoma viral oncogene homolog 1 (*AKT1*) with the generated genetic network in ST treated hASCs. *HSPB6* inhibits apoptosis of murine tumor cells and protects against oxidative damage (Ghulmiyyah et al., 2011; Chen et al., 2014), while *AKT1* helps mediate cell survival and clonogenic potential (Song et al., 2005; Xu et al., 2012; Gharibi et al., 2014; Liu et al., 2014). Supporting observations in ASCs, epithelial Caco-2 cells likewise show downregulation of apoptosis-related genes in response to treatment with ST. Comparison of the apoptosis-regulating TNF/FasL pathway in ST-infected hASCs and Caco-2 cells reveals similarities in cellular responses regarding survival pathways across the two distinct cell types [Supplementary Figure S2].

**FIGURE 5:**
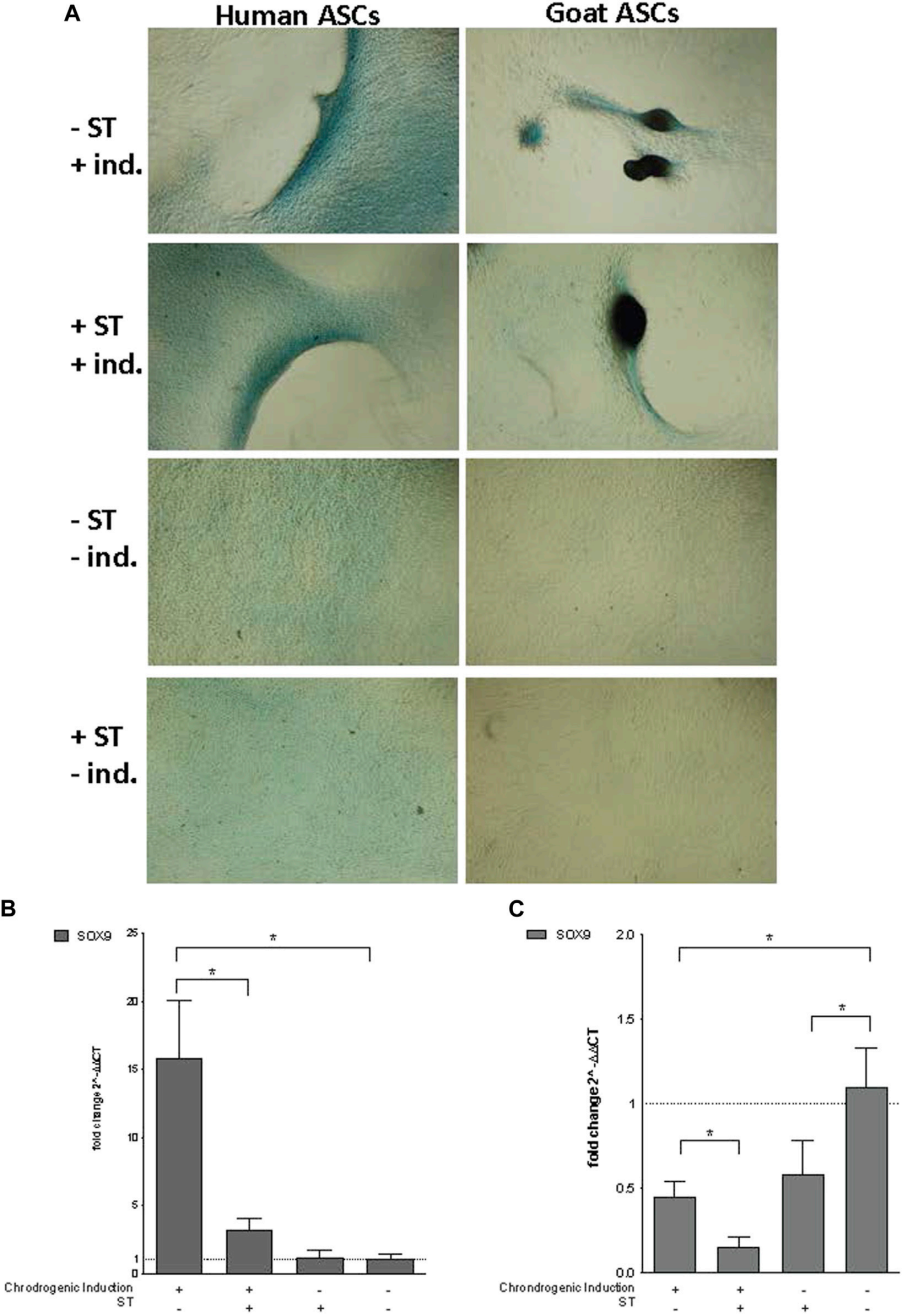
Chondrogenic induction. Representative images are shown in phase contrast at ×40 magnification. **(A)** Alcian Blue staining of chondrogenic differentiation in ASCs post-microbial association. Human and goat ASCs were cultured in chondrogenic differentiation medium for 14 days, and subsequently stained with Alcian Blue. Cellular condensation, as well ridge and micromass formations that stain positive were observed in human and goat ASCs induced for chondrogenesis, independent of S.T treatment. Some background staining was observed in S.T-treated and non-treated cells cultured in control medium, but cells remained in monolayer. Expression of chondrogenic markers post-microbial association was analyzed *via* quantitative PCR analysis of SOX9 expression in **(B)** human and **(C)** goat ASCs induced with chondrogenic differentiation medium and/or treated with S.T.

Upregulated in ST-treated hASCs were genes involved in molecular organization, differentiation, and proliferation (Figure 5). Epidermal differentiation influencer patched 2 (*PTCH2*) displayed increased levels of expression in ST-treated hASCs. The upregulation of *PTCH2* in conjunction with the connection to Hh signaling in the network suggests promotion of hASC proliferation pathways in response to co-incubation with ST (Adolphe et al., 2014). Immunomodulatory regulator, central regulator superoxide dismutase 3 (*SOD3*), was induced in treated hASCs (Figure 3), aligning with previous observations in INFγ/LPS-activated microglial cells (Kemp et al., 2010). Extracellular superoxide dismutase (EC-SOD) facilitates bacterial clearance and an anti-inflammatory response by promoting phagocytosis (Bowler et al., 2004; Kim et al., 2011; Manni et al., 2011). Upstream regulators of *SOD3*, *SOX10* and heparin sulfate (HS), were predicted *via* MAP to have increased activity in treated hASCs. Both *SOX10* and heparin sulfate (HS), are known to play a role in the maintenance of multipotency and self-renewal (Shakhova and Sommer, 2008; Helledie et al., 2012). Other regulators of *SOD3*, interferon gamma (*IFNG*), *IKBKB*, and *NOS3,* also had a predicted increase in activity for infected hASCs. *INFG* activation is of particular interest given an *INFG*-activated MSC suppresses T-cells and provide the necessary signal for MSC immunosuppression (Sivanathan et al., 2014).

As with the apoptotic pathways mentioned above, the immunomodulatory response of epithelial Caco-2 cells was evaluated in comparison to that of hASCs. Interestingly, while Caco-2 cells strongly induced TLR signaling in response to exposure to *Salmonella*, which facilitates downstream cellular response to pathogenic challenges, this observation was not seen in the challenged hASCs [Supplementary Figure S2]. Interestingly, this indicates that hASCs do not use TLR signaling in response to *Salmonella* association, suggesting an alternative mechanism for *Salmonella* internalization that is cell death independent.

## Analysis of trilineage differentiation post-microbial association

### Chondrogenic differentiation

Co-incubation with ST did not decrease the ability of ASCs to undergo chondrogenesis. Differentiated ST-treated hASCs migrated to form ridges that stained with Alcian Blue (Figure 5A). Differentiated ST-treated gASCs displayed more advanced morphological changes compared to hASCs (Figure 5A). After ridge formation, gASCs aggregated and formed clumps that also stained with Alcian Blue. For comparison, uninfected and uninduced control cells remained in a monolayer and exhibited minimal background staining.

Essential for this cartilage formation in ASCs is SRY (sex determining region Y)-box 9 (*SOX9*) (Bi et al., 1999), which encodes a transcription factor that promotes cartilage-specific extracellular matrix components (Bell et al., 1997; Han and Lefebvre, 2008). Expression of *SOX9* in infected hASCs and gASCs was measured by qPCR 14 days post chondrogenic induction. Infected and induced hASCs had decreased *SOX9* expression (*p* = 0.04) compared to non-infected but induced hASCs, while non-induced hASCs showed no significant change in *SOX9* expression (Figure 5B). Chondrogenic induction of non-infected hASCs increased *SOX9* expression compared to hASCs treated with non-inducing control medium (*p* = 0.034). gASCs displayed different expression patterns of *SOX9* across treatment types as compared to hASCs (Figure 5C). Induced gASCs, both infected and non, displayed decreased *SOX9* expression as compared to non-induced controls (*p* = 0.012). ST-treated gASCs, both induced and non, also had decreased *SOX9* expression as compared to non-induced and non-treated gASCs (*p* = 0.027, *p* = 0.039 respectively). Overall, hASCs had increased expression of *SOX9* as compared to gASCs, but no clear pattern of effect of infection status or induction status on *SOX9* expression across the two cell types emerged during the treatment.

### Adipogenic differentiation

**FIGURE 6:**
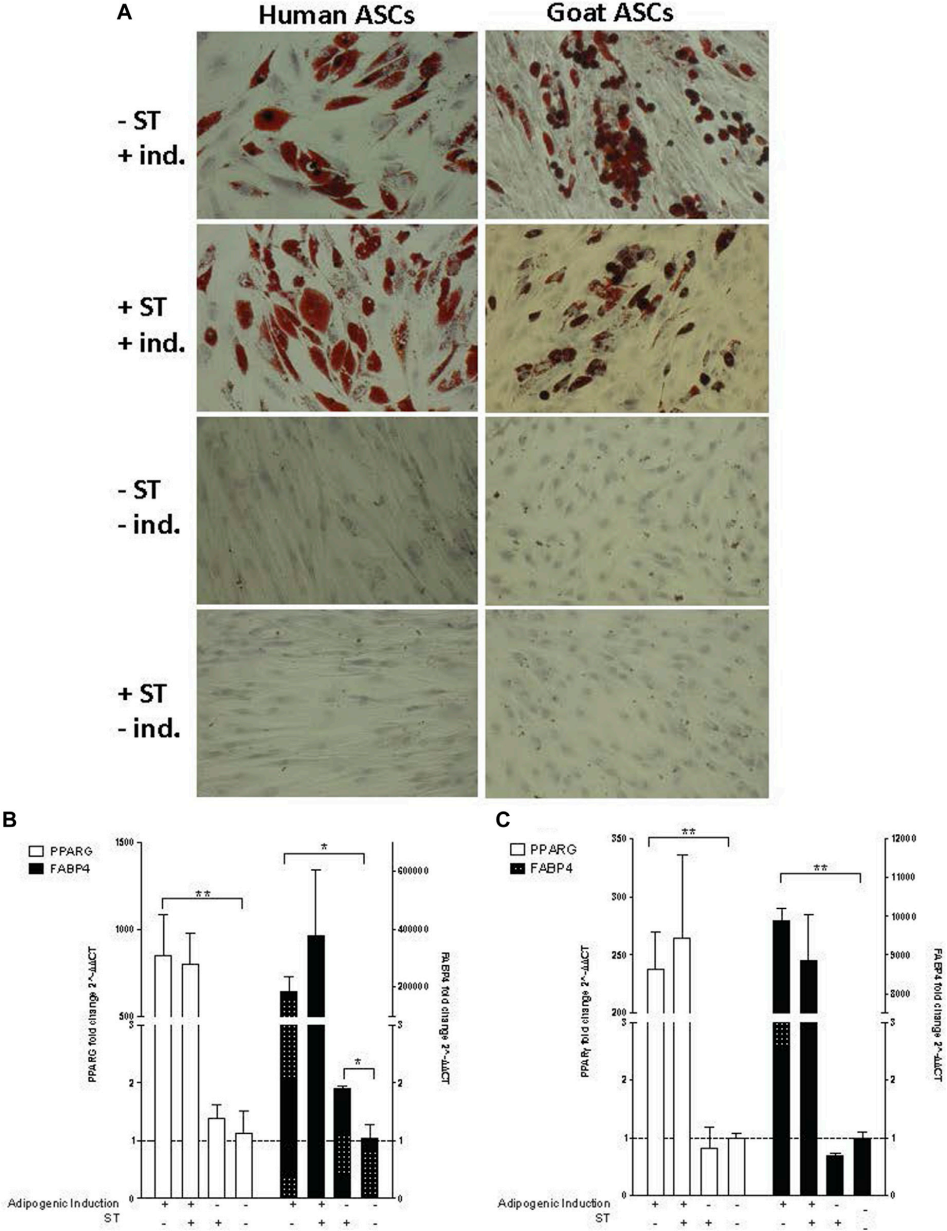
Adipogenic induction. Representative images are shown in bright field at ×200 magnification. **(A)** Oil Red O staining of adipogenic differentiation in ASCs post-microbial association. hASCs and gASCs were cultured in adipogenic induction medium for 21 days and stained with Oil Red O. Accumulation of cytoplasmic lipid droplets were observed in ASCs induced for adipogenesis, independent of S.T treatment. S.T-treated and non-treated ASCs cultured in control medium did not yield lipid-positive cells. Expression of adipogenic markers in ASCs post-microbial association was analyzed *via* quantitative PCR analysis of PPARγ and FABP4 expression in **(B)** human and **(C)** goat ASCs induced with adipogenic induction medium and/or treated with S.T. Data is presented as fold change (±SEM) relative to expression levels in non-treated, non-induced cells (fold change ∼1, indicated by the dotted line). Statistical significance of *p <* 0.05 is denoted by an asterisk (*), and *p* < 0.01 denoted by two asterisks (**).

Infected and non-infected ASCs cultured in adipogenic medium accumulated lipid-filled vacuoles that stained with Oil Red O (Figure 6A). Cells not cultured in adipogenic medium, uninduced ASCs, did not form these lipid-filled adipocytes and did not stain with Oil Red O. No visual morphological differences were observed between non-induced ST-treated cells and non-induced control cells.

The lack of observed visual differentiation was further explored *via* the measurement of *PPARG* and fatty acid binding protein 4 (*FABP4*) expression, which together indicated induction of early events within adipogenesis (Shin et al., 2009; Shi et al., 2013). *PPARG* and *FABP4* expression was measured by qPCR in cells 21 days post induction. ST treatment alone did not have a marked effect on *PPARG* or *FABP4* expression in either hASCs or gASCs, instead, induction status had a more distinct effect on expression (Figures 6B, C). Significant increases in *PPARG* expression were observed in both human and goat cells grown in induction medium as compared to uninduced controls (*p ≤* 0.0001, *p ≤* 0.0001, respectively).

The expression of *FABP4*, a fatty acid binding protein specific to mammalian adipose tissue (Bernlohr et al., 1985; Bakhtiarizadeh et al., 2013), followed the same trend as *PPARG* across both hASCs and gASCs. No significant difference was observed between induced ASCs treated with ST and uninfected control cells. Non-induced hASCs, but not gASCs, treated with ST has a significant increase in *FABP4* expression (*p =* 0.019). Both species had increased *FABP4* expression in cells cultured in adipogenic induction medium (*p =*0.029, *p ≤* 0.0001) (Figures 6B, C).

### Osteogenic differentiation

**FIGURE 7:**
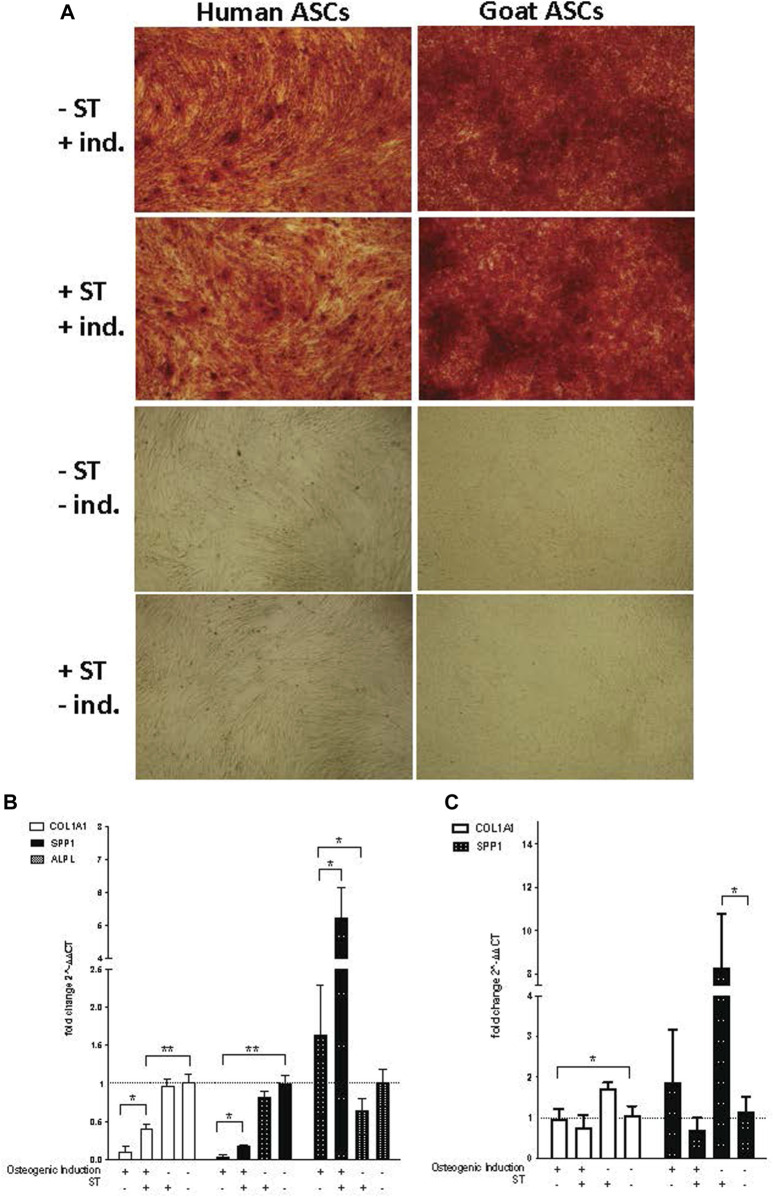
Osteogenic induction. Representative images are shown in phase contrast at ×40 magnification. **(A)** Alizarin Red S staining of osteogenic differentiation in ASCs post-microbial association. hASCs were cultured in osteogenic differentiation medium for 14 days, whereas gASCs for 21 days and stained with Alizarin Red S. ASCs cultured in osteoinductive medium stained positive for calcium but did not stain when cultured in control medium, regardless of S.T treatment. Expression of osteogenic markers in ASCs post-microbial association were analyzed *via* quantitative PCR analysis of COL1A1, ALP and OPN gene expression in **(B)** human and **(C)** goat ASCs induced with osteogenic differentiation medium and/or treated.

Osteogenic induction of infected ASCs resulted in the formation and accumulation of mineralized calcium deposits within the monolayer, as confirmed by Alizarin Red S staining [Figure 6A]. There was no readily apparent visual difference between induced cells treated or not treated prior with ST. ASC controls grown in expansion medium did not display the same calcium mineralization as their induced counterparts and did not stain with Alizarin Red S, independent of ST treatment (Figure 7A).

The expression of osteogenic-related genes, collagen type I alpha 1 (*COL1A1*), alkaline phosphatase (*ALPL*) and *SPP1,* was evaluated by qPCR at the termination of differentiation. *COL1A1* encodes for a major component of the most abundant collagen found in bone matrix (Viguet-Carrin et al., 2006) and so gene expression of *COL1A1* was used as a marker of osteogenic differentiation in ASCs. *COL1A1* expression was significantly decreased in induced cells as compared to non-induced cells, regardless of ST treatment status (*p ≤* 0.0001) (Figures 7B, C). Across induced hASCs, *COL1A1* expression was significantly higher in ST-treated cells compared to un-infected controls (*p* = 0.025). No difference in expression was detected between non-induced infected and non-infected control hASCs. gASCs displayed no significant change in *COL1A1* expression across induced cells that were either treated with ST or not, though there was a significant decrease in *COL1A1* expression by induced gASCs as compared to uninduced controls (*p* = 0.018) (Figure 7C).

*ALPL* expression, which is responsible for the availability of phosphate ions during the production of bone mineral during matrix maturation (Tenenbaum and Heersche, 1982; de Bernard et al., 1986; Choi et al., 1996), was measured across both cell types in response to induction and infection status. Expression of *ALPL* was significantly higher in induced hASCs than in non-induced cells, regardless of ST treatment (*p* = 0.02) (Figure 7B). ST-treated induced hASCs displayed 3.3-fold higher *ALPL* expression than non-ST treated but induced hASCs (*p* = 0.03). Non-induced hASCs showed no significant difference in expression patterns by ST-treatment status. *ALPL* expression was not detected in any gASCs across all treatment types.

Expression of a third osteogenic-related gene, *SPP1*, was evaluated in ASCs in response to induction and ST-treatment. *SPP1* is a non-collagenous bone protein expressed during the mineralization phase late in osteogenesis (Nagata et al., 1991). *SPP1* expression was repressed in both ST-treated and non-treated induced hASCs (*p ≤* 0.0001) (Figure 7B). ST-treated and induced hASCs displayed 4.3-fold higher expression of *SPP1* than induced but not infected cells (*p* = 0.002). Non-induced hASCs showed no difference in expression between infected and non-infected cells, though expression of *SPP1* was generally increased in comparison to the induced hASCs. No significant difference was detected between ST-treated and non-treated induced gASCs, however; there was a significant change in *SPP1* expression observed in non-induced cells across ST-treatment status (Figure 7C). A 7.2-fold increase in *SPP1* expression was observed in non-induced but ST-treated gASCs as compared to non-induced, non-infected cells (*p ≤* 0.05).

**FIGURE 8:**
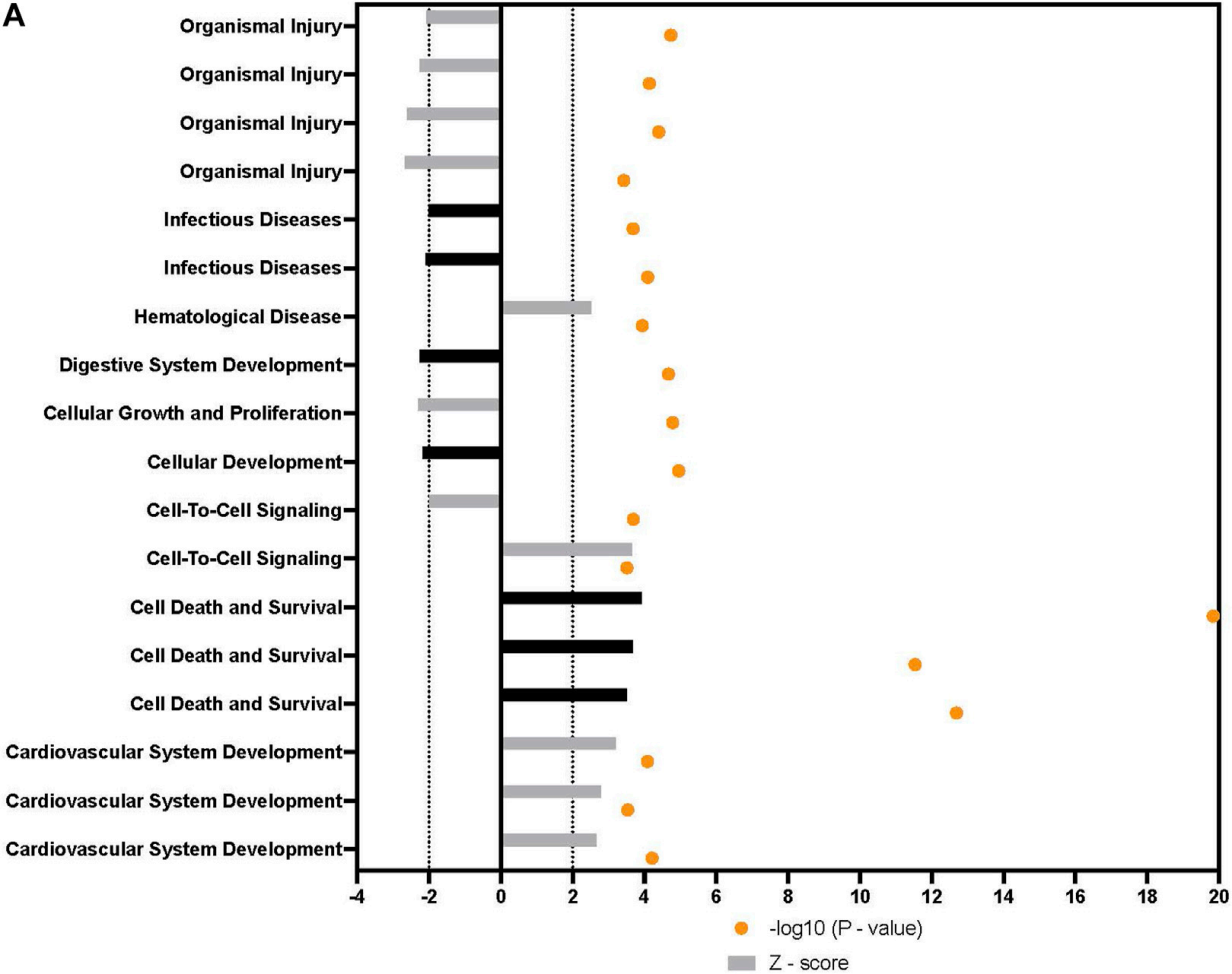
Downstream trends analysis of differentially expressed genes in hASCs induced towards osteogenesis post microbial challenge. The IPA regulation z-score algorithm was used to identify biological functions expected to increase or decrease based on the gene expression changes observed in our dataset. Predictions base on *p*-value and z-score; positive z-score implies an increase in the predicted function, a negative z-score a decrease (z-score ≥ 2 or ≤ −2 represented by black dotted lines). *p*-values ≤0.05 (orange dots determined by Fischer’s exact test), illustrate a significant association between a given biological function and genes differentially expressed in our dataset (*p*-value ≤0.05). Shapes associated with each gene name and broad category indicate the general classification of the gene product, enzyme, growth factor, etc.

**FIGURE 9:**
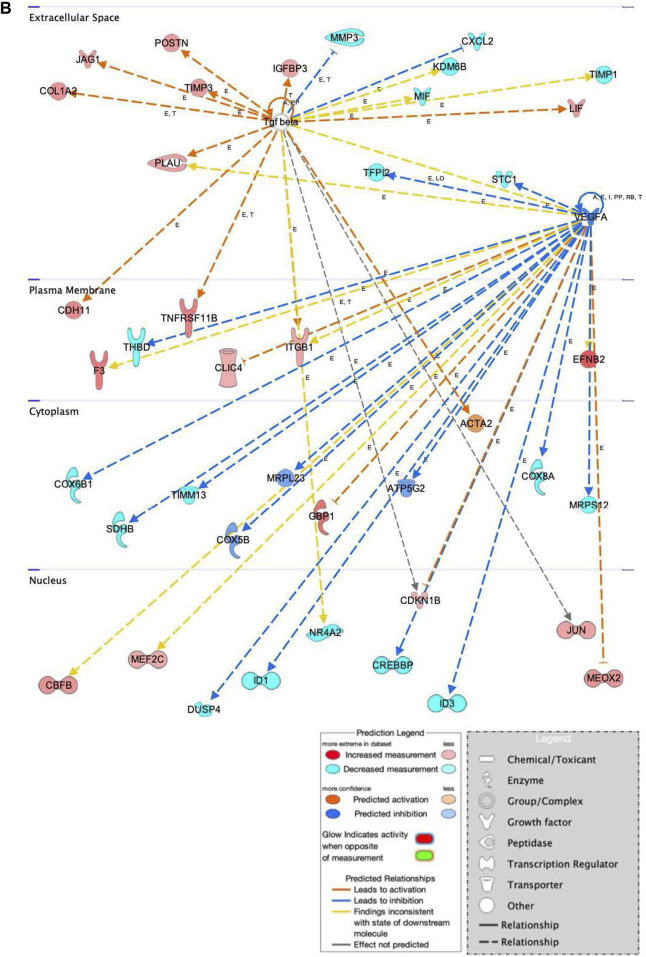
Network displays interactions between genes involved in cellular movement, hematological system development and function, and inflammatory response that were differentially expressed in hASCs induced towards an osteogenic lineage following S.T challenge. Upregulated genes are colored in shades of red, downregulated in shades of green. Genes in white were inserted by IPA because they are connected to this network; dashed and solid lines denote indirect and direct relationships between molecules. The IPA molecule activity predictor assessed the activity of molecules strongly connected to this network; blue and orange colored molecules are predicted to have decreased and increased activity, respectively. Shapes associated with each gene name and broad category indicate the general classification of the gene product, enzyme, growth factor, etc.

In addition to the specific markers of osteogenesis laid out above, a broader survey of gene activity of hASCs in response to osteogenic induction and ST treatment was done utilizing RNAseq. Pretreatment of hASCs with ST, followed by 14 days of osteogenic induction, resulted in the differential expression of 1,060 genes (data not shown). Downstream biological functions of these differentially expressed genes were determined using RNAseq z-score algorithm in IPA. These expression data predicted the repression of genes associated with cell-to-cell signaling, inflammation, and the response to infectious disease (*p*-value ≤0.05, z-score ≥2) (Figure 8). Genes involved in cellular communication, migration, and lineage commitment were also differentially expressed in ST-treated and induced hASCs. Differentially expressed genes related to the pathways mentioned above include stanniocalcin 1 (*STC1*) and mesenchyme homeobox 2 (*MEOX2*) (Figure 9). *STC1* expression, a response to apoptotic signals (Kim et al., 2013) that is involved in inflammation suppression and mineral homeostasis (Block et al., 2009; Kim et al., 2013), was downregulated in differentiated and ST-treated hASCs. In contrast, *MEOX2* was upregulated in the infected and induced hASCs. Also upregulated in ST-treated osteogenic differentiated hASCs (Figure 9), was chloride intracellular channel 4 (*CLIC4*), which is induced during cellular stress and influences cell cycle arrest and apoptosis (Suh et al., 2005). Intracellular chloride regulates cation transport and may be involved in cellular signaling and *CLIC4* expression has reportedly been associated with Ca^2+^-induced differentiation of keratinocytes (Suh et al., 2005; Suh et al., 2007).

Genes related to bone anabolism were also differentially expressed in ST-treated hASCs. Bone anabolism, an important part of osteogenic differentiation, is in part regulated by the Wnt/β-catenin signaling cascade. The Wnt/β-catenin signaling cascade is influenced by extracellular factors, including heparin sulfate proteoglycans (Ling et al., 2009), and regulated in part by periostin (*POSTN*) (Bonnet et al., 2012; Cho et al., 2012). Increased induction of *POSTN* following ST challenge and osteogenic differentiation was observed as compared to cells not challenged with ST (Figure 9). Another Wnt activator, secreted frizzled-related protein 1 (*SFRP1*), had predicted activation in the ST-treated hASCs, and related transcription factors *JUN* and *AXIN2* were upregulated in differentiated and challenged ASCs [Supplementary Figure S3]. The Wnt/β-catenin canonical pathway overall appears to be activated by ST treatment and drives MSCs towards pluripotency [Supplementary Figure S3].

Osteogenic commitment is regulated by multiple genes in MSCs. Upregulated in this dataset were the ephrin-B2 ligand (*EFNB2*) and *EPHB4* receptor (Figure 9). *EFNB2* is involved in osteogenic commitment and is required for the differentiation of osteoclasts and osteoblasts *in vivo* (Tierney et al., 2013). Both the *EPHB4* receptor and *EFNB2* ligand are reportedly expressed on the surface of MSCs (Tierney et al., 2013). Also involved in proliferation and osteogenesis is *COL1A2*, the increased expression of which was also detected in ST treated hASCs (Figure 9). *COL1A2* promotes cellular proliferation and osteogenesis, a response in part regulated by ERK/AKT1 pathway activation (Tsai et al., 2010).

The progression of osteoblasts is driven by activation of ERK mitogen-activated protein kinase family (*MAPK*), which phosphorylates related transcription factors (Salasznyk et al., 2004). Gene expression data from ST challenged hASCs indicated there was upregulation of integrins involved in *MAPK1* activation, as well as activation of intracellular signal transducer, phosphatidylinositol-4,5-bisphosphate 3-kinase (*PI3K*) [Supplementary Figure S4].

Genes related to immunomodulatory behaviors were also detected as differentially expressed in this dataset of ST-treated and differentiated hASCs (Figure 9). One immunomodulatory related MSC gene, cadherin 11 (*CDH11*), showed increased expression in treated hASCs. *CDH11* expression is known to be upregulated by TGFB and subsequently, increases calcium-dependent cell-to-cell interactions in MSCs (Park et al., 2014). Though *CDH11* engagement on fibroblast-like synoyiocytes (FLS) has been reported to produce inflammatory mediators IL6 and IL8 (Park et al., 2014), differential expression of these cytokines was not detected in this transcriptomic dataset. The association with ST did overall alter the genetic activity of MSCs in osteogenically induced cells, diverging from the lack of visual cues indicating ST treatment had no effect on differentiation. At the level of gene expression, ST treatment did alter the behavior of induced MSCs towards pro-proliferation pathways.

## Discussion

Host-microbe interactions are important for the immune system, cellular development, and the expansion of metabolic capabilities (Visconti et al., 2019; Gomaa, 2020). The presence of microbes on host tissues is an essential part of the host defense repertoire against pathogens (Kim et al., 2017), and the interaction between microbes and host cells is known to be important for cellular proliferation and development (Furusawa et al., 2013). Though many microbe-host relationships are beneficial to host health and function, pathogenic microbes present currently unknown short and long-term consequences to proximal host cells (O’Rourke and Kempf, 2019). Many stem cells are primed for bacterial interactions and even take cues from association with commensal bacteria (Nigro and Sansonetti, 2015; Ferguson et al., 2021), but the unique proliferation and differentiation properties of stem cells may also make them a target for pathogens looking to evade the immune system and persist long-term (Granick et al., 2012; Jain et al., 2020).

MSCs are one host cell type whose role in responding to inflammation and infections means interactions with pathogenic microbes are common occurrence (Brandau et al., 2014; Mezey, Nemeth, 2015). In light of their known immunomodulatory role *in vivo*, MSCs have been investigated as potential therapeutics in instances of drug-resistant pathogens (Yuan et al., 2014; Johnson et al., 2022). (Yuan et al., 2014) illustrated the ability of bone marrow-derived MSCs to increase clearance of methicillin-resistant *S. aureus* (MRSA) in a rat model. Further, work by Maiti and colleagues showed that MSC stimulation with MRSA not only resulted in changes to cell proliferation but also the induction of inflammatory markers (Maiti and Jiranek, 2014). MSCs may also act as a potential treatment for the most drug resistant mycobacterial pathogen, *Mycobacterium abscessus* (Kim et al., 2016). The potential application of MSCs in these pathogenic settings makes it imperative there is a thorough understanding of the effect of pathogens on MSC cellular activity.

*Salmonella*, the most common intestinal pathogens, is the leading causes of foodborne illness (Wilson et al., 2020). The prevalence of *Salmonella* as a pathogen, and specifically as an inflammation-causing intracellular pathogen (Pilar et al., 2013), makes it an important organism to study in the context of stem cell activity. In this study, ASCs were vulnerable to microbial infection *in vitro* with multiple *Salmonella* serovars, suggesting pathogen susceptibility may be a common characteristic, especially when considered in conjunction with the observations of Kol et al. (2014). This investigation sought to expand on the results of Kol et al. (2014) and to provide an insight into the specific effect *S. enterica* ssp *enterica* serotype Typhimurium LT2 invasion may have on the distinct immunomodulatory behaviors and differentiation activity of human and goat adipose-derived MSCs.

Important to understanding the results found here is the co-incubation method used in this study. Other studies have made use of microbe-associated molecular patterns (MAMPs) like LPS or long-term, continuous exposure to heat-inactivated pathogens to study the stem cell response to infectious conditions (Brandau et al., 2014; Ti et al., 2015; Huang et al., 2020). In this study, the use of viable bacteria and short exposure time may better mimic the *in vivo* physiological conditions in which MSCs interact with infiltrating microbes. MSCs must migrate to sites of inflammation (Liu H et al., 2018), where bacteria may be transient, leaving only a narrow window of opportunity for direct MSC-microbe interaction. The goat and human MSCs utilized in this study were infected with and internalized ST within 60 min of co-incubation. Broadly, these ST challenged MSCs had altered expression of prototypical genes markers for inflammatory responses, apoptosis, and differentiation.

Responding to sites of inflammation is a key role of MSCs (Liu H et al., 2018). During the course of infection, *Salmonella* initiates epithelial inflammation and the rapid induction of pro-inflammatory cytokines *via* calcium-mediated activation of NFKB1 (Eckmann et al., 1993; Gewirtz et al., 2000). *NFKB1* expression was not altered by ST treatment in this study, but treatment with LPS did result in a significant increase in expression for gASCs. NFKB1 is a central regulator of innate and adaptive immune responses and plays a key role in the induction of inflammation (Liu et al., 2017). NFKB1 proteins are available and inactive; activity depends on phosphorylation-dependent degradation of NFKB1 inhibitors, thus the lack of change in mRNA expression is not unexpected (Oeckinghaus and Ghosh, 2009). It is possible the expression of *NFKB1* was transiently increased in response to ST co-incubation, but then expression waned as other immunomodulatory genes were activated.

Out of the seven immunomodulatory markers surveyed in this study, IL8 was the most significantly altered in both human and goat ASCs as a response to ST co-incubation, confirming previous observations (Gewirtz et al., 2000). IL8 can be rapidly expressed and secreted by multiple cell types and is utilized as a clinical biomarker of inflammation (Bernhard et al., 2021). Previous studies showed a dose-dependent increase in IL8 by human bone marrow-MSCs in response to LPS treatment (Rougier et al., 1998) and by human intestinal epithelial stem cells in response to dietary compound forskolin (Wang et al., 2018). IL8 is an important cytokine for the recruitment of neutrophils to sites of inflammation, but elevated levels of circulating IL8 in cancer patients are associated with poorer health outcomes (Matsushima and YangOppenheim, 2022). In conjunction with this finding, IL8 has also been shown to have stimulatory effects on stem cells, encouraging proliferation and differentiation related to malignant tumor growth (Matsushima and YangOppenheim, 2022). In the context of these previous findings, the increased production of IL8 as a response to ST treatment in the stem cells of this study is a potentially concerning result. The association of increased IL8 with more negative host health outcomes indicates *Salmonella* infection may drive host stem cells towards more harmful outcomes, though these findings are preliminary and require follow-up work to confirm in an *in vivo* setting.

Another pleiotropic cytokine involved in innate tissue response to injury and maintenance of undifferentiated MSC status is IL6 (Tie et al., 2019). In this study, a significant increase in *IL6* expression was observed in LPS-treated gASCs. A similar trend was noted in ST-treated gASCs and hASCs, although this change was not statistically significant. *IL6* expression is known to decrease during osteogenic differentiation (Pricola et al., 2009). While mature osteoblasts display enhanced osteogenic differentiation, primitive MSCs experience a decrease in proliferation following IL6 treatment (Cho et al., 2007). This implies that the influence of IL6 on osteogenesis is complex and dependent on the differentiation status of targeted MSCs (Cho et al., 2007). The divergent responses of MSCs to IL6 by cellular status suggests stem cell response to cytokine-promoting infections, like ST, changes the secretion of small molecules that are capable of crosstalk between inflammatory and differentiation pathways.

Exposure to MAMPs alone, without an associated viable pathogen, is sufficient to influence ASC signaling. The MSC response to these microbial components is mediated by Toll-like receptors (TLRs); the hASCs used in this study are known to express *TLR1-6* and *TLR9* (Hwa Cho et al., 2006). LPS, a key component of the *Salmonella* cell wall (Hoshino et al., 1999), is a TLR4 agonist. LPS has been shown to influence osteogenesis in hASCs and BM-MSCs by increasing mineralization, ALP activity, and expression of osteogenic markers (Hwa Cho et al., 2006; Cho et al., 2010; Raicevic et al., 2012; Fiedler et al., 2013). These LPS-induced changes in differentiation may be in part mediated by TLRs expressed on MSCs and dependent on the aforementioned NFKB1 activation (Rougier et al., 1998; Bernhard et al., 2021). The overall pattern of immunomodulatory gene expression differed between the human and goat cells, indicating a potential difference in activity between these two groups. Goat cells may be a more easily accessible sample type, but the heightened expression of immunomodulatory markers in response to LPS in comparison to the human MSCs is an area that requires further exploration if goat MSCs are to act as model for human regenerative medicine.

While the conditions of this study did not lead to an observed induction of TLR gene expression in MSCs, previous reports highlight the role of TLR activation in MSC physiology. In non-induced mouse BM-MSCs, TLR2 activation inhibited spontaneous adipogenic differentiation and increased osteogenesis, but inhibited trilineage differentiation in induced cultures (Pevsner-Fischer et al., 2007). Osteogenic markers in non-induced ST-treated ASCs were upregulated in our study, supporting these previous observations in BM-MSCs. Furthermore, TLR-activated MSCs recruit immune cells; TLR-activated macrophages secrete oncostatin M, a cytokine that induces osteogenesis and inhibits adipogenesis in BM-MSCs (Guihard et al., 2012). Providing further evidence for a link between microbe induced TLR signaling and osteochondrogenic pathway induction are the diminished capabilities of myeloid differentiation primary response 88 (*MYD88*) deficient MCSs. MSCs deficient in *MYD88*, crucial for TLR signaling (Kawai et al., 1999), lack both osteogenic and chondrogenic potential (Pevsner-Fischer et al., 2007), supporting the interconnectedness of MSC immunomodulatory and differentiation activity.

Broader examination of the transcriptome of ST treated hASCs in this study suggests a physiological shift in favor of cell survival and proliferation. Previous studies have confirmed *Salmonella* is able to exist intracellularly long term, evading the host defense through shelter in immune-privileged cells (Ross et al., 2014; Goldberg et al., 2018). Caco2 cells in this study displayed a marked upregulation in genes regulating the apoptotic pathway when exposed to ST, while the infected hASCs showed no difference in the expression of these same genes. Under oxidative stress, like that surrounding an ST infection, MSCs display a reduced ability to repair tissue and an increased propensity towards senescence (Zhu et al., 2006; Kim et al., 2010). These stress conditions decrease the MSC capacity for osteogenesis in favor of adipogenic commitment (Kim et al., 2010). Through upregulation of redox mediators, hASCs respond to and mitigate oxidative stress, helping to ensure cell viability, multipotency, and promote immune suppression (Hu et al., 2018). As versatile immune privileged cells, MSCs presented with a microbial challenge may function as a safeguard by generating an anti-inflammatory environment, creating an atmosphere conducive for infection clearance by resident phagocytes (Evans et al., 2021). Results of this study indicate ST infection may induce the expression of immunomodulatory genes in MSCs, but more interestingly indicate that ST association decreases apoptotic responses, ultimately driving MSC towards proliferation, differentiation, or senescence rather than cell death. However immuno-privileged stem cells may be, it is unlikely that the hASC response to infection is without physiological consequences. The influence of inflammatory mediators, which come about in response to such an infection, on lineage commitment appears to prime hASCs towards a pro-osteogenic phenotype.

ST treatment of hASCs had a significant effect on osteogenic differentiation. Consistent with findings by Fiedler et al. (2013), there was an observed increase in *ALPL* expression for osteogenically-induced and ST-treated hASCs. Increased *ALPL* expression is also consistent with previous work showing increased expression in LPS-treated hASCs at 10 days post-osteogenic induction (Hwa Cho et al., 2006). gASCs in this study did not have any detectable *ALPL* expression. Cells were harvested 21 days post-induction, when the mineralization phase was likely occurring (Owen et al., 1990), and *ALPL* expression at that point may have already decreased (Owen et al., 1990; Choi et al., 1996). *ALPL* expression is a marker of early osteogenesis and MSCs lacking this crucial gene experience alterations in their cellular fate (Liu W et al., 2018). While differentiation stage is a known effector of *ALPL* expression, cell density is also factor (Mao et al., 2016). (Mao et al., 2016) illustrated *ALPL* expression was increased in higher density stem cell environments, indicating cell-to-cell signaling is also an important regulator of differentiation status. *ALPL* is a key marker of osteogenesis, but consistent trends were not seen across the human and goat cells or across treatments in this work. The lack of this early osteogenic marker may be attributable more to the time of collection rather than an actual deficiency of *ALPL* expression overall.

Coinciding with the lack of early osteogenic marker *ALPL*, upregulation of *SPP1* (a late marker of osteogenesis) (Owen et al., 1990; Cowles et al., 1998), was observed in ST-treated gASCs. *SPP1* was not significantly upregulated in induced and ST-treated cells; it is possible that the osteoinductive effect of ST-treatment in induced ASCs was masked, as the medium contained additives that already strongly induce osteogenesis. In addition to osteogenic commitment, the 6-glycosylated phosphoprotein SPP1 has a significant role in cellular stress and immunity. SPP1 is an inflammatory mediator and is reported to have anti-inflammatory effects in acute colitis (Wang and Denhardt, 2008) and SPP1-deficient mice have an impaired ability to clear *Listeria monocytogenes* (Ashkar et al., 2000). Conversely, in chronic disease states increased *SPP1* expression is thought to have the opposite effect, as it decreases survival time for patients with lung cancer and is associated with increased Irritable Bowel Disease symptoms (Miao et al., 2021; Nguyen et al., 2022). The observation in this study of increased *SPP1* expression in ST-treated but uninduced gASCs implies a complex microbial-dependent response in MSCs. Taken along with previous findings and the other results of this study, the upregulation of *SPP1* in response to microbe-induced inflammation may drive affected MSCs towards osteogenic commitment.

Differential markers of expression following ST treatment were observed in non-induced ASCs. We observed a concomitant decrease in chondrogenic differentiation in response to ST treatment, as illustrated by a decrease in *SOX9* expression in ASCs. SOX9 is required for commitment to chondrogenic lineage (Akiyama, 2008). To our knowledge, this is the first report on the direct effect of bacterial association on MSC chondrogenesis. Osteogenic markers were observed to increase, important to note as osteogenesis and chondrogenesis are tightly coupled processes (Joyce et al., 1990), both are regulated by proteins in the TGFβ superfamily (Wan and Cao, 2005). Though, an inverse relationship between osteogenic and chondrogenic differentiation has been demonstrated, where microRNAs targeting genes important for osteogenesis were upregulated during chondrogenesis and *vice versa* (Suomi et al., 2008). The demonstrated differential regulation of trilineage differentiation markers in non-induced but pathogen-treated cells highlights the potential direct influence of microbes on ASC lineage commitment.

ST association in this study was shown to impact key modulators of both apoptosis and trilineage differentiation in adipose-derived MSCs. Treatment with the intracellular pathogen ST resulted in increased expression of pro-proliferative MSC pathways and of mineralization related *SPP1*. Pathways dictating the response of MSCs to injury, microbial products, and inflammation intersect with those regulating cellular differentiation, providing a route for pathogen influence on lineage commitment. However, the extent to which pathogens can influence MSC differentiation and the mechanisms responsible for this potential control have yet to be fully described, though it is clear that microbial reprogramming of host cells is a possible consequence of association. With MSCs poised as a potential therapeutic in regenerative medicine, where pathogen association is a likely factor, the consequences of pathogenic interactions on MSC activities must be further investigated for safe application and use.

## Data Availability

The data are available on NCBI in the 100K Pathogen Genome BioProject assession number of PRJNA239251.
