# Supplementary figures and images for: Salmonella enhances osteogenic differentiation in adipose-derived mesenchymal stem cells
Source: Front Cell Dev Biol. 2023 Mar 15;11:1077350. doi: 10.3389/fcell.2023.1077350 (PMC10055666; doi:10.3389/fcell.2023.1077350)

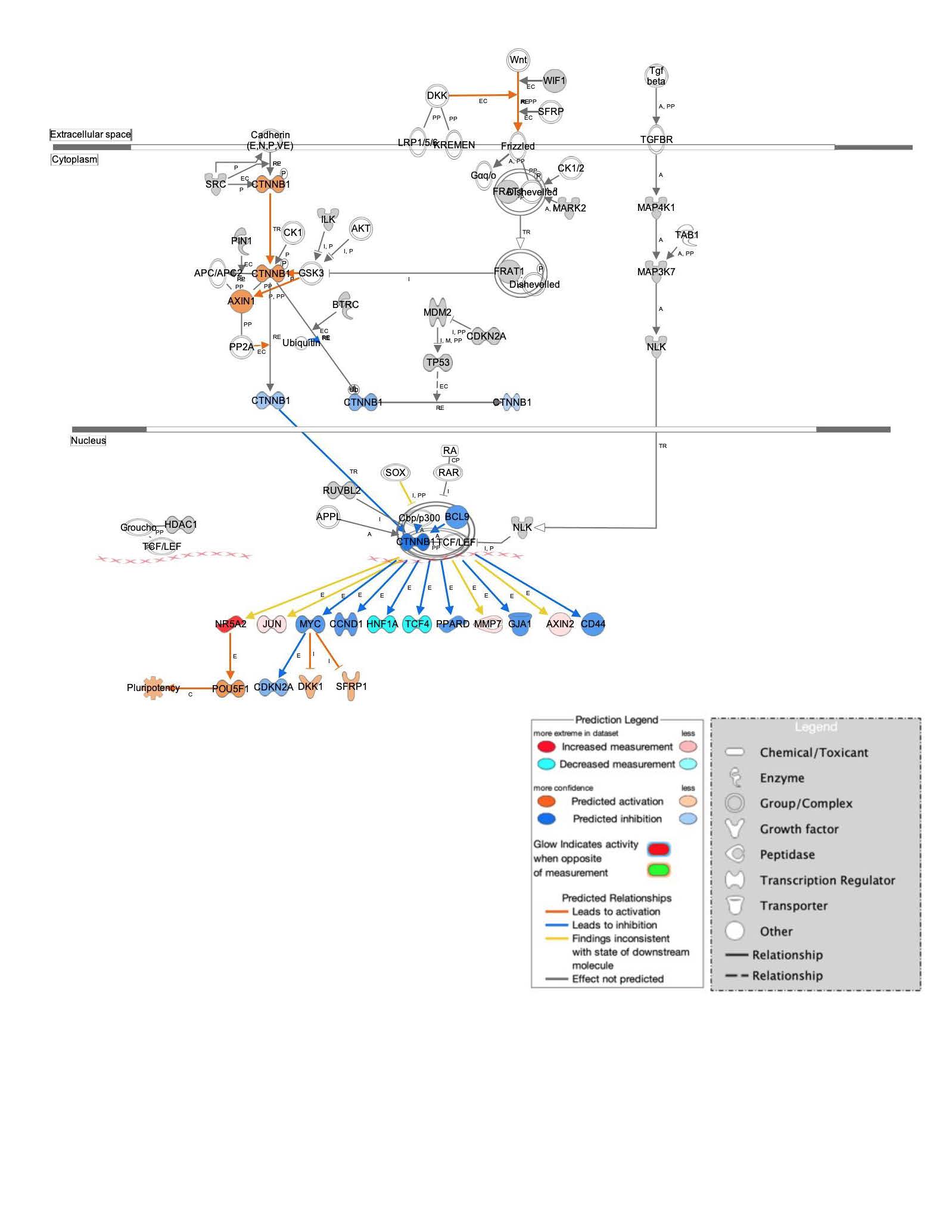

Supplement: Supplementary file 1 [file Image3.JPEG]

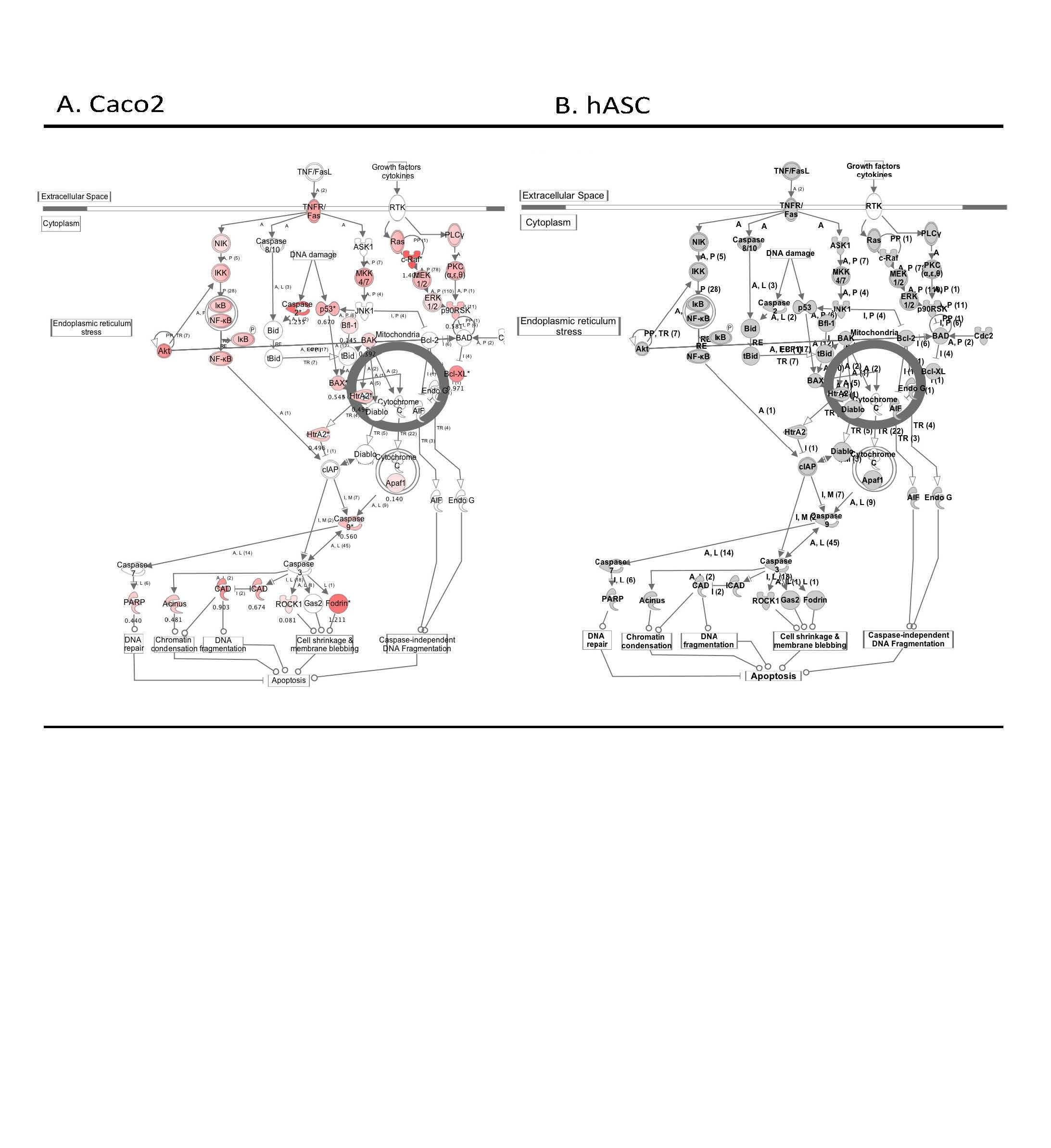

Supplement: Supplementary file 2 [file Image1.JPEG]

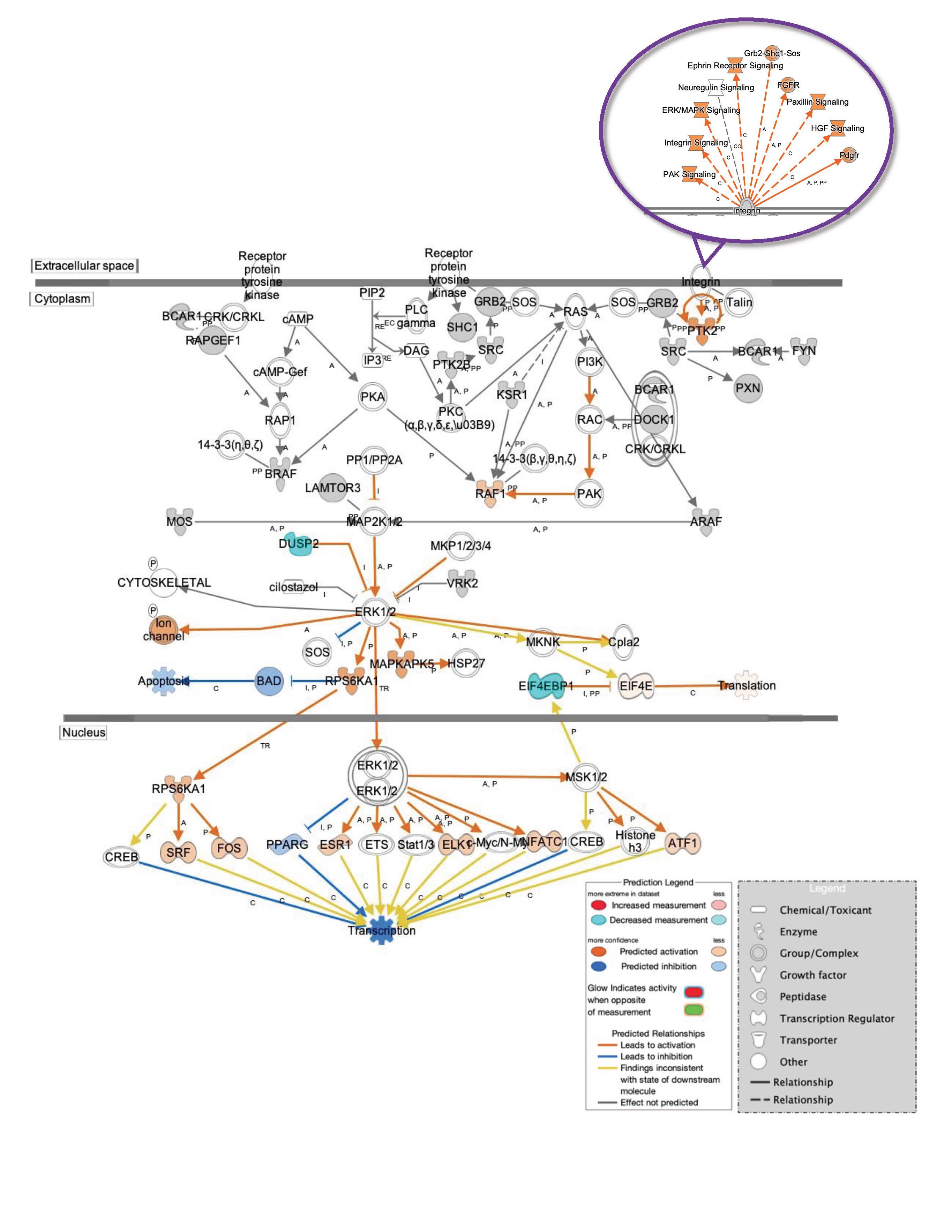

Supplement: Supplementary file 3 [file Image4.JPEG]

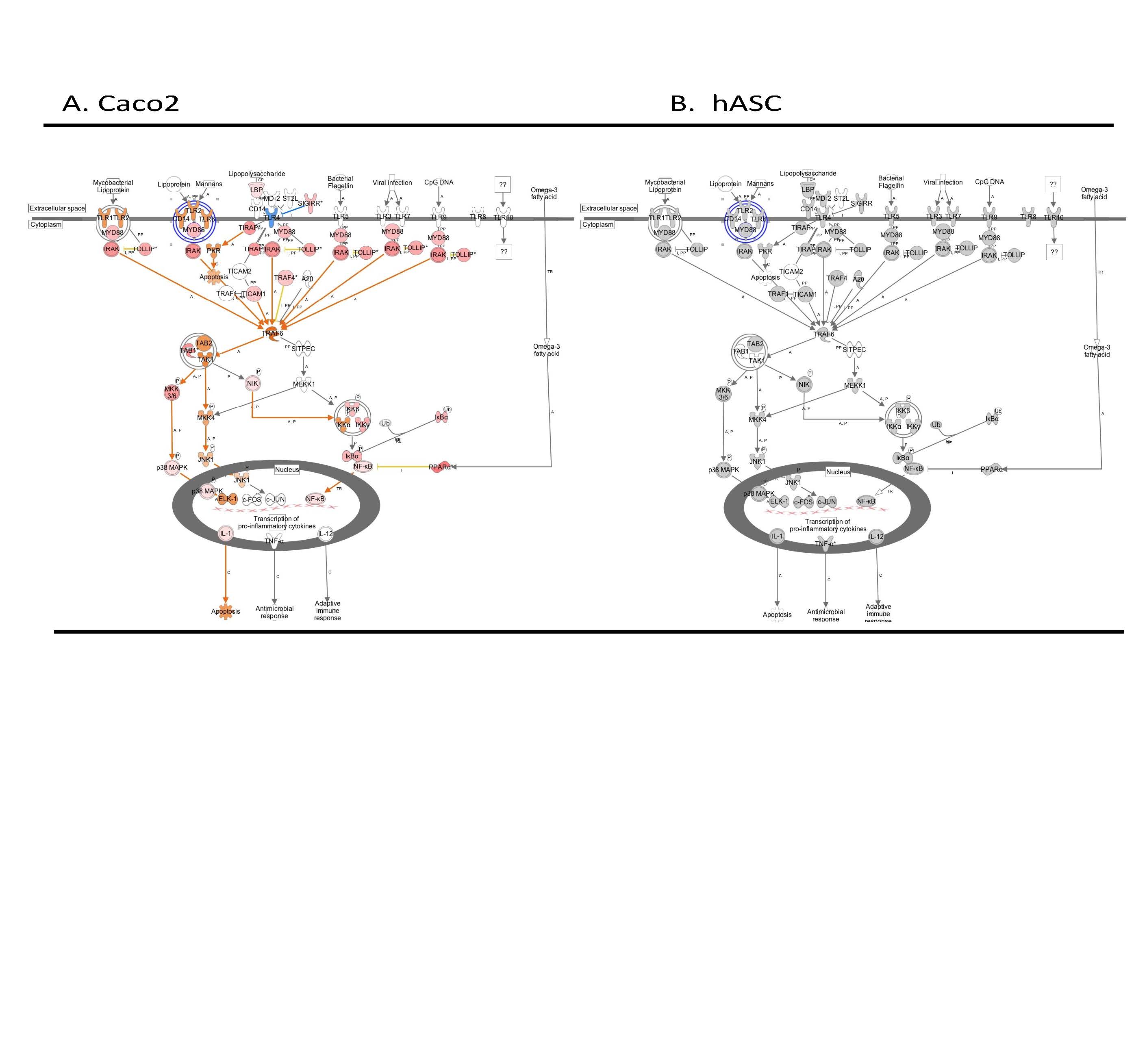

Supplement: Supplementary file 4 [file Image2.JPEG]
